# Discriminating Different Bladder and Bladder Outlet Dysfunctions by Urinary Biomarkers in Women with Frequency–Urgency Syndrome

**DOI:** 10.3390/biomedicines11030673

**Published:** 2023-02-23

**Authors:** Jia-Fong Jhang, Yuan-Hong Jiang, Hann-Chorng Kuo

**Affiliations:** Department of Urology, Hualien Tzu Chi Hospital, Buddhist Tzu Chi Medical Foundation, Tzu Chi University, Hualien 97004, Taiwan; alur1984@hotmail.com (J.-F.J.); redeemerhd@gmail.com (Y.-H.J.)

**Keywords:** overactive bladder, frequency–urgency syndrome, biomarker, cytokine

## Abstract

Objectives: To investigate the role of urinary biomarkers in discriminating different bladder and bladder outlet dysfunctions in women with frequency–urgency syndrome. Materials and Methods: Urine samples collected from 146 women with frequency–urgency syndrome and 34 controls were investigated. All patients were included in previous clinical trials of functional urology studies and underwent a videourodynamic study. Patients with frequency–urgency syndrome were subdivided into idiopathic detrusor overactivity (IDO), neurogenic detrusor overactivity (NDO), dysfunctional voiding (DV), and hypersensitive bladder (HSB) subgroups. Urine samples were collected before any treatment, and urinary inflammatory proteins (interleukin- (IL-) 1β, IL-2, IL-6, IL-8, tumor necrosis factor-α (TNF-α), and vascular endothelial growth factor (VEGF)), neurogenic proteins (nerve growth factor (NGF), brain-derived neurotrophic factor (BDNF), and prostaglandin E2 (PGE2)), and oxidative stress biomarkers (8-isoprostane, total antioxidant capacity (TAC), and 8-hydroxydeoxyguanosine (8-OHdG)) were measured and compared between the different OAB subgroups and controls. Results: Of the 146 patients, 31 had IDO, 41 had NDO, 45 had DV, and 29 had HSB. The control group included 34 women. The patients with HSB had lower urinary TAC and IL-2 levels than the controls. The patients with IDO, NDO, and DV had significantly higher urinary TNF-α levels than those with HSB. The patients with IDO and NDO showed an increase in the urinary 8-isoprostane levels, whereas the patients with IDO had higher urinary IL-2, NGF, and BDNF levels than those with NDO. The other urinary inflammatory biomarkers did not show enough significant differences to discriminate between the different bladder and bladder outlet dysfunctions. Conclusions: The urinary levels of inflammatory, neurogenic, and oxidative stress biomarkers varied widely among the patients with bladder and bladder outlet dysfunction. This study’s results provide evidence that women with frequency–urgency syndrome and different urodynamic subtypes have varying bladder inflammation and oxidative stress conditions, which might have an impact on treatment outcomes.

## 1. Introduction

Frequency–urgency syndrome is a common condition with a high prevalence rate globally [1,2]. When there is no definite pathology that can be found, an overactive bladder (OAB) is defined. The International Continence Society defines OAB as a symptom complex characterized by urgency, with or without urgency incontinence, usually with frequency and nocturia [3]. OAB is a collection of symptoms of frequency and urgency without an identifiable pathophysiology and might be related to bladder oversensitivity, low bladder compliance, or detrusor overactivity (DO) [4]. In fact, not all patients with frequency–urgency syndrome can be satisfactorily treated with medication, and about 30% of patients need additional therapies, including detrusor botulinum toxin A injection, percutaneous nerve stimulation, sacral nerve neuromodulation, or invasive surgical intervention [5]. The unclear pathophysiology and incorrect subgrouping of frequency–urgency syndrome are the causes of medical treatment failure [6]. The possible disorders for frequency–urgency syndrome refractory to medical treatment include an occult neurogenic bladder, undetected bladder outlet obstruction (BOO), urethral incompetence-related frequency–urgency syndrome, the aging process, urothelial dysfunction, chronic bladder ischemia, chronic bladder inflammation, central sensitization, and autonomic dysfunction [7]. Therefore, a correct diagnosis is essential for successfully treating patients with frequency–urgency syndrome.

Women with frequency–urgency syndrome might have both storage and voiding lower urinary tract symptoms (LUTS). However, an accurate diagnosis of lower urinary tract dysfunction based on women’s LUTS is not usually reliable. To obtain a precise diagnosis and treatment strategy, a videourodynamic study (VUDS) is considered to be an essential tool for investigating bladder and bladder outlet dysfunctions in women whose frequency–urgency symptoms cannot be relieved after initial medical treatment [8]. VUDS provides an accurate diagnosis. However, its use is limited due to its invasive nature and radiation exposure. Urinary biomarkers have been proposed as non-invasive tools for diagnosing different urological disorders [9]. Recent studies have gradually differentiated bladder-centered and non-bladder-centered frequency–urgency syndrome and interstitial cystitis/bladder pain syndrome (IC/BPS) using a cluster of urinary biomarkers, including inflammatory proteins, neurogenic proteins, and oxidative stress biomarkers [10,11]. This study aimed to identify different VUDS-confirmed bladder and bladder outlet dysfunctions in women using urinary biomarkers. We hypothesized that these bladder and bladder outlet dysfunctions could be accurately clinically diagnosed using a non-invasive tool, and the urinary biomarker levels might provide the underlying pathophysiology of frequency–urgency syndrome and treatment guidance without using VUDS.

## 2. Materials and Methods

### 2.1. Patients and Urine Samples

This study included 146 women with frequency–urgency syndrome and 34 controls. A total of 180 urine samples collected from patients with different bladder and bladder outlet dysfunctions and controls in previous functional bladder studies were investigated. The urine samples were collected over the last 2 years. All patients had storage symptoms such as frequency, nocturia, urgency, and urgency urinary incontinence with or without voiding symptoms. Urine samples were collected at baseline before the clinical trials. The control women were patients with genuine stress urinary incontinence undergoing anti-incontinence surgery. The control women did not have frequency–urgency symptoms. All patients underwent VUDS at baseline with the following findings: idiopathic DO (IDO, *n* = 31), neurogenic DO (NDO, *n* = 41), dysfunctional voiding (DV, *n* = 45), hypersensitive bladder (HSB, *n* = 29), and VUDS-proven normal urinary tract function (controls, *n* = 34). The diagnosis of the different bladder and bladder outlet dysfunctions was in accordance with the International Continence Society recommendations [12]. Patients with NDO had neurological diseases such as cerebrovascular accident, Parkinson’s disease, and early dementia confirmed by neurologists. Patients with spinal cord injury or multiple sclerosis, suspicions of IC/BPS, prominent urethral stricture, and severe disability to bladder emptying were excluded from the study. All patients had previously participated in different clinical trials for the diagnosis or treatment of functional bladder dysfunction and had agreed to have their urine samples saved for future clinical studies within a 10-year span. Furthermore, they were informed about the purpose of the study. This study was approved by the Institutional Review Board and Ethics Committee of the Hualien Tzu Chi Hospital (approval number: IRB 111-102-B, dated 17 May 2022). The informed consent requirement was waived due to the retrospective nature of the analysis. To obtain a significant statistical level of 0.05 and an effective size of 0.8, a total patient number of 60 was needed to achieve a power of 0.90 between the experimental group and the control group.

### 2.2. VUDS Procedure

VUDS was performed using standard procedures with a 6-French dual-channel catheter to measure intravesical pressure and an 8-French rectal balloon catheter to record the intra-abdominal pressure. The detrusor pressure (Pdet) was obtained by subtracting the intra-abdominal pressure from the intravesical pressure (Pves). Pelvic floor muscle electromyography (EMG) was recorded concomitantly using surface EMG patches placed at the perianal area. Normal saline containing 20% urografin was infused into the bladder at a rate of 20–30 mL/min. After catheterization and draining of the residual urine, patients were examined in a sitting position. The filling cystometry and pressure flow studies, Pves, Pdet, sphincter EMG activity, and voiding cystourethrography (VCUG) with C-arm fluoroscopy were also recorded. Furthermore, the following VUDS parameters were measured: the first sensation of bladder filling (FSF), full sensation (FS), urge sensation (US), bladder compliance, maximum flow rate (Qmax), voiding Pdet at Qmax (Pdet.Qmax), voided volume, corrected Qmax (Qmax/voided volume^1/2^), post-void residual volume (PVR), cystometric bladder capacity (CBC), voiding efficiency (VE, defined as voided volume/CBC × 100), bladder contractility index (BCI, defined as Pdet + [5 × Qmax]), and urethral sphincter EMG activity [8,11].

All descriptions and terminologies used in the clinical symptom recording and VUDS were according to the recommendations of the International Continence Society [12,13]. Patients with normal bladder sensation, no phasic detrusor contraction during the filling phase, CBC > 350 mL, normal Pdet.Qmax (<30 cmH_2_O) or low Pdet.Qmax, but Qmax > 15 mL/s and VE > 90%, and adequate relaxation of the urethral sphincter on EMG activity during the voiding phase were considered urodynamically normal [14]. Patients with an increased bladder FSF, FS, or US during repeat VUDS, but with neither definite DO or urgency sensation nor bladder outlet dysfunction being noted, were classified as having urodynamic HSB [15].

DO was defined as urodynamic evidence of spontaneous detrusor contractions occurring either during bladder filling (phasic DO) or before uninhibited detrusor contraction voiding upon reaching bladder capacity (terminal DO) [13]. Patients were diagnosed with detrusor underactivity if they did not have voiding detrusor contractility of >10 cmH_2_O and needed to void by abdominal straining or were unable to void [14]. Patients with frequency–urgency syndrome and urodynamic DO and having a history of cerebrovascular accident, parkinsonism, early dementia, or intracranial lesion were classified as having NDO. DV was defined as an intermittent or fluctuating flow due to intermittent involuntary contractions of the periurethral striated muscle during voiding in neurologically normal individuals [15]. DV is characterized by increased external sphincter activity during the voiding phase and a typical spinning-down shape in voiding cystourethrography during VUDS [11]. Patients were classified as having a non-relaxing sphincter if the urethral sphincter EMG activity increased during the filling phase but remained constantly high during the voiding phase. The urodynamic findings in each bladder and bladder outlet dysfunction and the control group are shown in Table 1.

### 2.3. Assessment of Urinary Biomarkers

The urinary biomarker assessments were performed in accordance with our previous study [16]. A total of 50 mL of urine samples was collected from the patients and controls before intravesical treatment or surgical interventions. The urine samples were obtained by self-voiding when patients had a full bladder sensation. Urine samples collected from patients with confirmed urinary tract infections were excluded. The samples were placed immediately on ice and transported to the laboratory. Next, they were centrifuged at 1800 rpm for 10 min at 4 °C. The supernatant was preserved in a freezer at −80 °C. The urine samples were centrifuged at 12,000 rpm for 15 min at 4 °C before further analyses were performed and were used for subsequent measurements of urinary cytokines, chemokines, and oxidative biomarkers.

### 2.4. Cytokine and Chemokine Assay

Inflammation-associated urinary cytokines and chemokines were assayed using commercial microspheres with a Milliplex^®^ Human cytokine/chemokine magnetic bead-based panel kit (Millipore, Darmstadt, Germany). A total of 9 targeted analytes, namely interleukin (IL)-1β, IL-2, IL-6, IL-8, tumor necrosis factor-α (TNF-α) (catalog number HCYTA-60K), vascular endothelial growth factor (VEGF), nerve growth factor (NGF), BDNF (catalog number HNDG3MAG-36K), and prostaglandin E2 (PGE2) (Cayman Chemical Co., Ann Arbor, MI, USA, No. 514010), were selected. These urinary proteins were selected based on previous studies of urinary biomarkers on OAB [16,17,18,19]. These analytes were measured using a multiplex kit (catalog number: HCYTMAG-60K-PX30). Urinary cytokine and chemokine level measurements were performed in accordance with the manufacturer’s instructions and methods used in previous studies [16,19]. A total of 25 μL of assay buffer, 25 μL of urine sample, and 25 μL of beads were added sequentially to 96-well plates (panel kits). The plates were incubated overnight in the dark at 4 °C. After removing the well contents and washing the plates twice with 200 μL of wash buffer, 25 μL of detection antibody was added to each well, and the plates were incubated in the dark on a shaker plate for 1 h at room temperature. A total of 25 μL of streptavidin–phycoerythrin solution was added to each well to form a capture sandwich immunoassay, and this was followed by incubation in the dark for 30 min at room temperature. Then, the well contents were removed, and the plates were washed twice with 200 μL of wash buffer. Finally, 150 μL of sheath fluid was added, and the plates were run on a MAGPIX^®^ instrument with the xPONENT^®^ 4.3 software. The median fluorescence intensity of each cytokine/chemokine target was recorded and analyzed to calculate the individual corresponding cytokine/chemokine concentrations in the urine samples.

### 2.5. Urinary Oxidative Stress Biomarker Assay

The quantification of 8-isoprostane, total antioxidant capacity (TAC), and 8-hydroxydeoxyguanosine (8-OHdG) in the urine samples was performed in accordance with the manufacturer’s instructions (8-OHdG ELISA kit, Abcam, Cambridge, MA, USA, ab285254; 8-Isoprostane ELISA kit, Enzo, Farmingdale, NY, USA, DI-900-010; and Total Antioxidant Capacity Assay Kit, Abcam, Cambridge, MA, USA, ab52635). The urinary biomarker assay was performed in accordance with our previous report [20].

The quantification of 8-isoprostane in the urine samples was performed in accordance with the manufacturer’s instructions (8-Isoprostane ELISA kit, Enzo). A total of 50 μL of 8-iso-PGF2α conjugation solution, 50 μL of 8-iso-PGF2α antibody solution, and 100 μL of the samples were sequentially added to 96-well plates (panel kits). The plates were incubated for 2 h at room temperature on a plate shaker at 500 rpm. The well contents were removed, and the plates were washed 3 times with 400 μL wash buffer. A total of 200 μL of p-nitrophenyl phosphate substrate solution was added to each well, and the plates were incubated for 45 min at room temperature without shaking. Finally, 50 μL of stop solution was added, and the plates were read immediately at 405 nm. The urinary 8-isoprostane levels were standardized based on the urinary creatinine levels measured using a commercial kit (Enzo Life Sciences Inc., Farmingdale, NY, USA, ADI-907-030A). The median fluorescence intensities of the targets were analyzed to calculate the corresponding concentrations of urinary biomarkers in the samples.

Quantification of TAC in the urine samples was performed. A total of 100 μL of copper (2^+^)-containing working solution and 100 of μL the samples were sequentially added to 96-well plates (panel kits). The plates were incubated for 90 min at room temperature on a shaker protected from light and evaluated using a microplate reader at 570 nm. The median fluorescence intensities of the targets were analyzed to calculate the corresponding TAC concentrations in the samples. Additionally, the quantification of 8-OHdG in the urine samples was performed. A total of 50 μL of biotin-detection antibody working solution and 50 μL of the samples were sequentially added to 96-well plates (panel kits). The plates were incubated for 45 min at 37 °C. The well contents were removed, and the plates were washed 3 times with 350 μL wash buffer. A total of 100 μL of HRP–streptavidin conjugate working solution was added to each well, and the plates were incubated for 30 min at 37 °C. The solution was discarded, and 350 μL wash buffer was used to wash the wells 5 times. A total of 90 μL of 3,3′,5,5′-tetramethylbenzidine substrate was added to each well, and incubation was then performed in the dark for 30 min at 37 °C. Finally, 50 of μL stop solution was added, and the plates were evaluated using a microplate reader at 450 nm.

### 2.6. Statistical Analysis

Continuous variables were expressed as means ± standard deviations, and categorical data were expressed as numbers and percentages. The urinary biomarker levels were compared among all the patient groups, IDO, NDO, DV, HSB, and controls. The differences between two subgroups were compared using post hoc tests based on the Mann–Whitney U test with the Bonferroni correction for controlling overall type-1 errors. Pearson’s correlation coefficients were calculated between the urine biomarkers and urodynamic parameters. The receiver operating curve (ROC) was performed, and the cut-off values (COV) of each urinary biomarker for HSB versus controls; IDO, NDO, and DV versus HSB; IDO and NDO versus DV; and IDO versus NDO were calculated. Urinary biomarkers with a mean value below the minimum detectable concentrations were not included in the final analysis. Statistical analysis was performed using the Statistical Package for the Social Sciences software for Windows version 20.0 (IBM Corp., Armonk, NY, USA), and *p*-values < 0.05 were considered statistically significant.

## 3. Results

Among the patients with IDO, NDO, DV, and HSB and the controls, the patients with NDO were older, and the patients with DV were younger. The DV subgroup showed significantly higher Pdet.Qmax values than the other subgroups. The IDO, NDO, and DV subgroups showed significantly smaller FSF, FS, and CBC levels than the controls. The HSB subgroup also showed smaller FS and CBC levels than the controls. All the subgroups showed lower compliances than the controls (Table 1).

Table 2 shows the urinary levels of inflammation-related biomarkers (IL-1β, IL-2, IL-6, IL-8, TNF-α, and VEGF), neurogenic proteins (NGF, BDNF, and PGE2), and oxidative stress biomarkers (8-isoprostane, TAC, and 8-OHdG) in all the groups. Among the subgroups, the IDO subgroup showed higher urinary VEGF and 8-isoprostane levels. The NDO subgroup showed significantly higher urinary TNF-α, PGE2, 8-isoprostane, and 8-OHdG levels. The DV subgroup showed lower urinary IL-2, VEGF, 8-isoprostane, and TAC levels than the IDO and NDO subgroups and higher urinary 8-OHdG levels than the HSB subgroup and the controls. The HSB subgroup showed similar urinary biomarker levels to the controls and lower urinary NGF and TAC levels than the controls. Table 3 shows the correlation between the urinary levels of each biomarker and urodynamic parameters. The inflammatory biomarkers (IL-1β, IL-6, IL-8, TNFα) and oxidative stress biomarkers (8-isoproatane and 8-OHdG) were significantly associated with a low voided volume, a small FS, or a small CBC.

The ROC was analyzed, and the COV and area under the curve (AUC) were calculated between the different subgroups to set the COV for each urinary biomarker in order to discriminate the different bladder and bladder outlet dysfunctions. The comparison of urinary biomarker levels between the different subgroups is shown in Table S1. The comparison between the HSB subgroup and the controls showed that only TAC showed an AUC ≥ 0.7 with satisfactory predictive values (COV ≤ 318.7, positive predictive value (PPV) = 85%, and negative predictive value (NPV) = 72.1%) in the HSB subgroup. When comparing the urinary biomarker levels between the IDO, NDO, and DV subgroups and the HSB subgroup, the only significant biomarkers were TNF-α (COV ≥ 1.035) and IL-2 (COV ≤ 0.390), with a high PPV but a low NPV. The comparison between the IDO and NDO subgroups and the DV subgroup showed that the urinary IL-1β (COV ≤ 0.695), IL-2 (COV ≥ 0.200), IL-6 (COV ≥ 0.885), VEGF (COV ≥ 0.908), BDNF (COV ≤ 0.510), and 8-isoprostane (COV ≥ 17.75) levels were higher in the IDO and NDO subgroups with AUC > 0.700 and PPV > 70%, but the NPV did not reach 70%. The comparison between the IDO and NDO subgroups showed that the urinary IL-1β (COV ≤ 0.510), IL-2 (COV ≥ 0.480), TNF-α (COV ≤ 1.460), NGF (COV ≥ 0.130), BDNF (COV ≥ 0.365), and TAC (COV ≥ 642.6) levels had AUC > 0.700, but only the urinary IL-1β, IL-2, TNF-α, NGF, and BDNF levels had PPV > 70% and NPV > 70% (Table 4).

**Figure 1:**
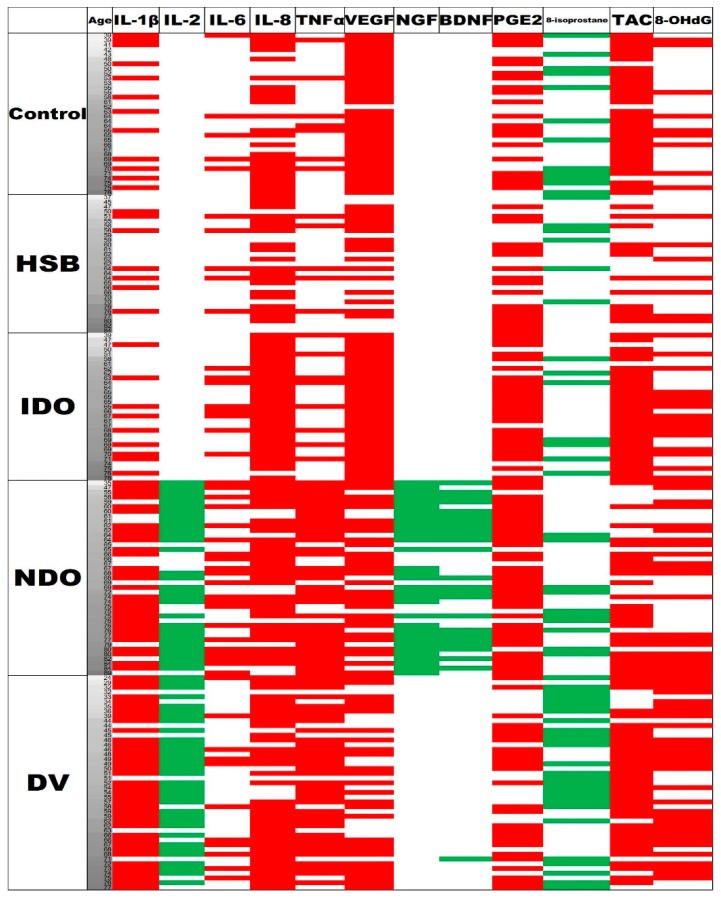
Clustered display of urinary biomarker levels with different videourodynamic study (VUDS) diagnoses by the respective cuff-off values between IDO + NDO + DV and HSB. Each row represents one patient. From top to bottom, the patients are listed in the order of controls (*n* = 34), HSB (*n* = 29), IDO (*n* = 31), NDO (*n* = 41), and DV (*n* = 45). Each column represents one urinary biomarker target. From left to right, the urinary biomarkers are listed in the order of inflammatory, neurogenic, and oxidative stress biomarkers. Red and green colors indicate the urinary biomarker levels with values higher and lower than the cut-off values of the biomarkers, respectively, in favor of bladder dysfunctions of IDO, NDO, and DV.

Figure 1 shows a clustered display of the urinary biomarker levels with different VUDS diagnoses (controls, HSB, IDO, NDO, and DV) by the respective cuff-off values between IDO + NDO + DV and HSB. The NDO and DV subgroups had more urinary biomarkers with higher levels than the HSB subgroup and the controls.

## 4. Discussion

This study’s results revealed that the urinary levels of inflammatory, neurogenic, and oxidative stress biomarkers varied widely among the patients with different bladder and bladder outlet dysfunctions. By analyzing the differences in urinary biomarker levels, the urinary biomarkers could be a useful diagnostic tool to differentiate bladder and bladder outlet dysfunctions, such as IDO, NDO, and DV, from HSB or the controls in women with frequency–urgency syndrome. Compared with the controls, the patients with HSB did not have an increase in oxidative stress biomarker levels, but they had higher urinary PGE2 and lower urinary TAC levels. The patients with higher urinary 8-isoprostane levels showed the presence of DO, either IDO or NDO. Higher urinary TNF-α and 8-OHdG levels indicated higher inflammatory and oxidative stress bladder conditions in the women with NDO and DV. These results proved that women with frequency–urgency syndrome and different bladder and bladder outlet dysfunctions have varying bladder inflammation and oxidative stress conditions.

The pathophysiology of frequency–urgency syndrome is multifactorial. Occult neurogenic bladder, undetected BOO, urethral-related dysfunctions, urothelial dysfunction with aging or diseases, chronic bladder ischemia, chronic bladder inflammation, central nervous system sensitization, and autonomic dysfunction are possible etiologies of refractory frequency–urgency syndrome [21]. The bladder urothelium and afferent nerves express transient receptor potential vanilloid receptor 1 and the purinergic receptor P2X3 [22,23]. These receptors are believed to be involved in the afferent pathways that control bladder sensation and urinary volume reflexes [24]. The increase in sensory receptor and functional protein expressions in the urothelium is highly associated with chronic inflammation of the bladder wall, such as in IC/BPS, DO, and BOO [25,26]. Without a bladder biopsy and immunohistochemistry staining, it will be difficult to differentiate which pathophysiology is involved in a woman with frequency–urgency syndrome. Because LUTS is unreliable in the diagnosis of bladder and bladder outlet dysfunctions, urinary biomarker levels might be able to provide objective evidence to discriminate patients with IDO, NDO, DV, and HSB.

In this study, we did not enroll patients with remarkable neurogenic lesions such as spinal cord injury, multiple sclerosis, transverse myelitis, etc. Instead, patients with urgency frequency symptoms and urodynamic DO and with a history of cerebrovascular accident, parkinsonism, early dementia, or intracranial lesion were classified as having NDO in this study. These patients might present with frequency–urgency symptoms but do not have other neurological signs. Urine biomarkers might be an early tool to detect the underlying neurological lesions.

Our previous studies on urinary biomarkers revealed that significantly higher levels of inflammatory cytokines in urine were noted in women with urinary tract infections and IC/BPS compared with controls [19,27]. The urinary oxidative stress biomarkers 8-OHdG and 8-isoprostane also showed a high diagnostic ability to distinguish the European Society for the Study of Interstitial Cystitis (ESSIC) type 2 IC/BPS patients from controls [20]. These urinary biomarker levels are likely to reflect the bladder inflammatory condition rather than the underlying histopathological status in patients with IC/BPS [28]. Urinary cytokine profiles are different between patients with ESSIC type 2 IC/BPS and OAB [16]. However, because several bladder and bladder outlet dysfunctions exist, differential diagnosis is necessary to further discriminate IDO, NDO, DV, and HSB.

In this study, we also found that the inflammatory and oxidative stress biomarkers were significantly associated with a small voided volume, small fullness sensation, and cystometric bladder capacity, suggesting that these biomarkers might have relationship with frequency–urgency syndrome and DO in patients with IDO, NOD, and DV. Previous studies demonstrated that both urinary 8-OHdG and IL-1β levels positively correlate with clinical symptoms in patients with DV. These findings also indicate that DV is associated with increased bladder inflammation through the induction of reactive-oxidative stress (ROS). Patients with DV who had successful treatment outcomes had significantly lower baseline urinary 8-isoprostane and TAC levels than those with unsuccessful outcomes [9]. These data suggest that urinary oxidative stress biomarkers might be associated with a higher grade of inflammation and mitochondrial dysfunction, resulting in poor treatment outcomes. In another study on patients with higher urinary PGE2 and BDNF levels, it was shown that detrusor underactivity might have a chance to recover bladder function compared with those with lower protein levels, suggesting that higher urinary levels of neurogenic proteins could indicate a higher grade of re-innervation in patients with an underactive bladder [29].

8-OHdG, 8-isoprostane, and TAC were applied as the oxidative stress and antioxidant biomarkers in BOO-related urinary tract dysfunction [30]. This study’s results demonstrated that, among the bladder and bladder outlet dysfunctions, the urinary 8-isoprostane and TAC levels were higher in the patients with IDO and NDO than in those with DV and HSB. The TNF-α and 8-OHdG levels were higher in the patients with DV than those in the patients with IDO or NDO. Both TNF-α and 8-OHdG were associated with increased intracellular ROS, resulting in downstream inflammation [31,32]. The increase in the urinary TNF-α and 8-OHdG levels suggests an increase in bladder inflammation in women with BOO due to DV. The urinary 8-OHdG, TNF-α, and PGE2 levels were significantly higher in the patients with IDO and DV than those in the controls [11]. TAC reflects the cumulative effect of all antioxidants from various endogenous anti-oxidative defense systems against harmful activities caused by oxidative stress [33].

The sensitivity and specificity of the other urinary biomarkers did not reach a satisfactory level in order to discriminate the different bladder and bladder outlet dysfunctions. In previous studies, urinary NGF, BDNF, and PGE2 were found to associate with OAB and antimuscarinic treatment outcome [34,35,36,37,38,39,40]. In this study, the urinary NGF levels in the patients with NDO and IDO were not significantly higher than those in the patients with HSB or controls. Although previous studies showed that NGF might be a potential biomarker to diagnose and predict the treatment outcomes of OAB [41], this study does not support this hypothesis, except for the fact that the urinary NGF levels in the patients with IDO were higher than those in the patients with NDO. The discrepancy of the sensitivity of urinary NGF in the diagnosis of different subtypes of frequency–urgency syndrome might be caused by the investigating kits, which might affect the testing results. In this study, the levels of another urinary biomarker, PGE2, which had been considered to be increased in patients with frequency–urgency syndrome due to neurogenic lesions and BOO [42,43], were found to be increased in the patients with IDO and HSB. Therefore, the elevated urinary PGE2 levels might indicate an increase in sensory innervation and be associated with a small bladder fullness sensation and bladder capacity.

A review of previous studies on urinary biomarkers in OAB showed a high diversity in the urinary levels of inflammatory and neurogenic biomarkers. Some inflammatory urinary interleukin levels were found to associate with worsening frequency–urgency symptoms, and some were lower in OAB patients [44]. It is possible that the underlying pathophysiology of frequency–urgency syndrome involves different grades of systemic or bladder inflammation, with or without neurogenic factors and BOO. Aging could also be another factor affecting urinary biomarker levels [45]. In addition, due to the lack of VUDS-verified bladder and bladder outlet dysfunctions, patients with frequency–urgency symptoms might be enrolled in an umbrella term as OAB, resulting in the different results in different studies. Changes in the amount of acetylcholine, adenosine triphosphate, and muscarinic receptors in the bladder tissue may be responsible for the development of OAB in older adults [46,47]. Recent studies hypothesized that chronic bladder ischemia, such as atherosclerosis, might play important roles in developing OAB and increasing sensory receptor expressions [48,49]. Ischemic bladders also showed elevated ROS and inflammatory factors, such as prostaglandins, in rabbit models [50,51]. In addition, oxidative stress was found to be important in the pathophysiology of OAB because the levels of 8-OHdG and malondialdehyde are increased [52]. Based on these findings, we included urinary oxidative stress biomarkers in this study and found elevated 8-isoprostane levels in patients with IDO and NDO and elevated 8-OHdG levels in the patients with DV, but not in the patients with HSB and the controls. Although the urinary TAC levels in the patients with IDO and NDO were similar to those in the controls, the urinary TAC levels were significantly lower in the patients with HSB, which could be used to discriminate IDO from HSB in women with frequency–urgency syndrome.

Our previous study revealed that serum C-reactive protein, NGF, IL-1β, IL-6, IL-8, and TNF-α were elevated in patients with OAB refractory to antimuscarinic treatment [53]. However, the midstream urinary biomarker levels were not correlated well with the serum level. In this study, TNF-α was the only biomarker that was significantly higher in the NDO subgroup than the other bladder and bladder outlet dysfunctions and controls, suggesting that the imbalance in the inflammatory proteins associated with the peripheral and central inflammatory pathway might be different in the pathogenesis of bladder and bladder outlet dysfunctions [54]. In our recent study, patients with DV had significantly higher urinary 8-OHdG, IL-1β, IL-8, and BDNF levels than controls. Both urinary 8-OHdG and IL-1β levels were positively correlated with clinical symptoms [9]. Single urinary biomarkers might not provide an accurate diagnosis of bladder and bladder outlet dysfunctions but might provide evidence for the underlying pathophysiology, which may help in deciding and selecting treatment modalities. It is also rational to use a cluster of urinary biomarkers to differentiate the underlying pathophysiology in women with frequency–urgency syndrome.

The strength of this study is that we investigated patients with VUDS and classified the bladder and bladder outlet dysfunctions according to the VUDS findings. This study’s results were more accurate than previous studies on urinary biomarkers in OAB, in which only symptoms were used to subgroup the patients into OAB dry and OAB wet. Another strength of this study is that oxidative stress biomarkers were added to the cluster of urinary biomarkers. The role of oxidative stress biomarkers in bladder and bladder outlet dysfunctions might be more important than inflammatory cytokines and neurogenic proteins in the pathogenesis of frequency–urgency syndrome. However, this study has several limitations, including a small number of patients, different age groups, no standardized procedure for urine sample collection, and the presence of medical comorbidities, which might have affected the urinary biomarker levels. Thus, future studies on urinary biomarkers are needed to correct these limitations.

## 5. Conclusions

This study’s results revealed that urinary TAC and IL-2 levels were lower in the patients with HSB than those in the controls. The patients with IDO, NDO, or DV had significantly higher urinary TNF-α levels than those with HSB. An increase in the urinary levels of the oxidative stress biomarker 8-isoprostane was noted in the patients with IDO and NDO, whereas the patients with IDO had higher urinary IL-2, NGF, and BDNF levels than the patients with NDO. The other urinary inflammatory biomarkers did not show enough significant differences to discriminate the different bladder and bladder outlet dysfunctions.

## Figures and Tables

**Table 1:** The videourodynamic parameters in patients with different bladder and bladder outlet dysfunctions and controls.

	IDO (*n* = 31)	NDO (*n* = 41)	DV (*n* = 45)	HSB (*n* = 29)	Control (*n* = 34)	*p*-Value	
Age	63.9 ± 8.96	68.9 ± 10.4	53.2 ± 14.2	63.0 ± 11.2	59.8 ± 11.1	<0.001	1 vs. 3, 2 vs. 35, 3 vs. 4
Pdet	18.0 ± 11.0	19.6 ± 11.8	47.8 ± 42.7	11.5 ± 9.49	15.1 ± 7.12	<0.001	1245 vs. 3
Qmax	16.1 ± 7.36	10.6 ± 7.06	10.6 ± 6.77	11 ± 6.69	18.9 ± 8.44	<0.001	1 vs. 234, 234 vs. 5
Volume	272 ± 134	176 ± 118	229 ± 116	232 ± 126	416 ± 152	<0.001	1234 vs. 5, 1 vs. 2
PVR	14.7 ± 40.8	116 ± 192	56.4 ± 66	75 ± 105	17.1 ± 71.9	0.002	1 vs. 3
FSF	109 ± 48.6	132 ± 74.4	125 ± 55.2	184.9 ± 207	170 ± 65.8	0.022	1 vs. 45, 123 vs. 4
FS	172 ± 74.2	196 ± 96.4	200 ± 82.5	243 ± 58.6	293 ± 95.8	<0.001	1235 vs. 4, 1234 vs. 5
Compliance	61.8 ± 42.9	62.9 ± 43.2	75.9 ± 83.8	98.4 ± 84.3	160 ± 101	<0.001	123 vs. 5
BCI	98.1 ± 36.4	72.4 ± 36.0	97.6 ± 51.5	65.3 ± 37.2	98.7 ± 50.4	0.010	135 vs. 4, 135 vs. 2
CBC	286 ± 135	288 ± 166	279 ± 134	302 ± 95.7	407 ± 166	0.001	1234 vs. 5
cQmax	0.98 ± 0.35	0.68 ± 0.50	0.64 ± 0.37	0.62 ± 0.35	0.88 ± 0.38	<0.001	234 vs. 1, 234 vs. 5
VE	0.95 ± 0.11	0.71 ± 0.36	0.8 ± 0.23	0.76 ± 0.34	0.96 ± 0.16	0.015	1 vs. 23, 23 vs. 5
AG number	−14.8 ± 20.1	−1.5 ± 19.4	24.9 ± 46.7	−11.7 ± 14.2	−22 ± 20.3	<0.001	1245 vs. 3, 23 vs. 5

Abbreviations: IDO: idiopathic detrusor overactivity; NDO: neurogenic detrusor overactivity; DV: dysfunctional voiding; HSB: hypersensitive bladder; Pdet: detrusor pressure (cmH_2_O); Qmax: maximum flow rate (mL/s); PVR: post-void residual volume (mL); FSF: first sensation of filling; FS: full sensation; BCI: bladder contractility index; CBC: cystometric bladder capacity; cQmax: corrected Qmax; VE: voiding efficiency; AG number: Abrams–Griffiths number.

**Table 2:** The urinary levels of inflammatory proteins, neurogenic proteins, and oxidative stress biomarkers in patients with different bladder and bladder outlet dysfunctions and controls.

	IDO (*n* = 31)	NDO (*n* = 41)	DV (*n* = 45)	HSB (*n* = 29)	Control (*n* = 34)	*p*-Value	Post Hoc
IL-1β	0.74 ± 0.9	1.28 ± 1.87	1.16 ± 1.4	0.71 ± 0.63	0.56 ± 0.26	0.192	
IL-2	0.74 ± 0.19	0.31 ± 0.23	0.28 ± 0.22	0.64 ± 0.14	0.79 ± 0.19	<0.001	234 vs. 1, 23 vs. 4, 234 vs. 5
IL-6	2.05 ± 2.62	4.28 ± 6.1	2.14 ± 5.16	1.53 ± 1.71	1.22 ± 1.29	0.588	2 vs. 5
IL-8	29.3 ± 58.8	37.0 ± 62.9	31.0 ± 63.9	48.3 ± 97.7	13.6 ± 22.8	0.451	
TNF-α	0.87 ± 0.4	2.42 ± 1.98	1.21 ± 0.33	0.92 ± 0.56	0.79 ± 0.31	<0.001	345 vs. 2, 25 vs. 3
VEGF	14.6 ± 5.96	11.4 ± 11.8	5.56 ± 4.91	8.44 ± 7.84	11.21 ± 5.3	<0.001	34 vs. 1, 15 vs. 3
NGF	0.26 ± 0.07	0.11 ± 0.08	0.21 ± 0.05	0.22 ± 0.07	0.27 ± 0.07	0.007	23 vs. 1, 1345 vs. 2, 234 vs. 5
BDNF	0.6 ± 0.22	0.37 ± 0.23	0.63 ± 0.15	0.74 ± 0.77	0.57 ± 0.14	0.629	135 vs. 2
PGE2	262 ± 175	481 ± 389	218 ± 187	283 ± 259	171 ± 107	0.047	135 vs. 2
8-isoprostane	32.5 ± 29.8	31.1 ± 28.2	12.9 ± 14.7	22.8 ± 17.3	17.5 ± 15.5	0.011	12 vs. 3
TAC	1558 ± 13,597	520 ± 452	604 ± 420	388 ± 279	1107 ± 1017	0.003	1 vs. 234, 24 vs. 5
8-OHdG	26 ± 17.7	27.1 ± 17.6	32.4 ± 19.4	18.4 ± 16.6	17.7 ± 13.6	0.001	45 vs. 2, 45 vs. 3

Abbreviations: IDO: idiopathic detrusor overactivity; NDO: neurogenic detrusor overactivity; DV: dysfunctional voiding; HSB: hypersensitive bladder; IL: interleukin; TNF-α: tumor necrosis factor-α; VEGF: vascular endothelial growth factor; NGF: nerve growth factor; BDNF: brain-derived neurotrophic factor; PGE2: prostaglandin E2; TAC: total antioxidant capacity; 8-OHdG: 8-hydroxydeoxyguanosine. All values of urinary biomarkers were corrected for creatinine.

**Table 3:** The correlation between urine biomarkers and urodynamic parameters.

	IL-1β	IL-2	IL-6	IL-8	TNFα	VEGF	NGF	BDNF	PGE2	8-Isoprostane	TAC	8-OHdG
Pdet (cmH_2_O)	0.125 *	−0.240 **	0.024	0.015	0.092	−0.139 *	−0.033	0.109	−0.038	−0.074	−0.017	0.044
Qmax (mL/s)	−0.095	0.102	−0.052	−0.069	−0.065	−0.012	−0.032	−0.061	−0.091	−0.040	0.000	−0.124 *
Volume (mL)	−0.144 *	0.112	−0.126 *	−0.139 *	−0.128 *	−0.099	−0.022	−0.034	−0.160 **	−0.128*	−0.051	−0.219 **
PVR (mL)	0.037	−0.151*	−0.012	0.051	0.059	0.026	0.010	0.083	0.080	0.006	−0.008	0.027
FSF (mL)	−0.075	−0.020	−0.095	−0.098	−0.014	−0.054	−0.013	−0.047	−0.052	−0.048	−0.085	−0.084
FS (mL)	−0.114	−0.024	−0.182 **	−0.121 *	−0.089	−0.083	−0.015	0.011	−0.092	−0.094	−0.101	−0.164 **
Compliance	−0.040	0.060	−0.032	−0.052	−0.049	−0.019	−0.028	−0.076	−0.044	−0.127 *	−0.113	−0.031
BCI	0.011	−0.056	−0.031	−0.038	0.016	−0.100	−0.049	−0.039	−0.067	−0.075	−0.002	−0.103
CBC (mL)	−0.105	−0.013	−0.141 *	−0.092	−0.070	−0.095	−0.015	0.020	−0.073	−0.116	−0.050	−0.198 **
cQmax	−0.044	0.097	0.016	−0.030	0.009	0.028	−0.043	−0.086	−0.049	0.003	0.047	−0.019
VE	−0.004	0.154 *	0.037	−0.037	−0.039	−0.079	−0.073	−0.086	−0.056	−0.033	0.012	−0.061
AG	0.154 **	−0.230 **	0.053	0.050	0.114	−0.096	−0.014	0.054	0.024	−0.042	−0.002	0.082

* *p* < 0.05, ** *p* < 0.005, Abbreviations: Pdet: detrusor pressure (cmH_2_O), Qmax: maximum flow rate (mL/s), PVR: post-void residual (mL), FSF: first sensation of filling, FS: full sensation, BCI: cladder contractility index, CBC: cystometric bladder capacity, cQmax: corrected Qmax, VE: voiding efficiency, AG number: Abrams–Griffiths number, IL: interleukin, TNF-α: tumor necrosis factor-α, VEGF: vascular endothelial growth factor, NGF: nerve growth factor, BDNF: brain-derived neurotrophic factor, PGE2: prostaglandin E2, TAC: total antioxidant capacity, 8-OHdG: 8-hydroxydeoxyguanosine.

**Table 4:** The cut-off value of urinary biomarkers in discriminating different bladder and bladder outlet dysfunctions and controls.

Urinary Biomarker Levels	HSB vs. Control	IDO + NDO + DV vs. HSB	IDO + NDO vs. DV	IDO vs. NDO
IL-1β	≥0.440	≥0.610	≤0.695 **	≤0.510 ***
IL-2	≤0.775 *	≤0.390 **	≥0.200 **	≥0.480 ***
IL-6	≥0.385	≥1.650	≥0.885 **	≤1.600
IL-8	≥93.295	≥1.860	≥7.505	≥3.710
TNF-α	≥0.770	≥1.035 **	≥1.545	≤1.460 ***
VEGF	≤3.410	≥3.235	≥9.080 **	≥7.850
NGF	≤0.245	≤0.115	≤0.125	≥0.130 ***
BDNF	≤0.415	≤0.310	≤0.510 **	≥0.365 ***
PGE2	≥180.605	≥133.01	≥185.24	≤626.195
8-isoprostane	≥22.610	≤10.19	≥17.75 **	≥7.115
TAC	≤318.7 ***	≥316.32	≥1203.74	≥642.6 *
8-OHdG	≤14.775	≥24.13	≤23.59	≤38.155

* AUC > 0.700; ** AUC > 0.700, and PPV > 70%; *** AUC > 0.700, PPV > 70%, and NPV > 70%. Abbreviations: IDO: idiopathic detrusor overactivity; NDO: neurogenic detrusor overactivity; DV: dysfunctional voiding; HSB: hypersensitive bladder; IL: interleukin; TNF-α: tumor necrosis factor-α; VEGF: vascular endothelial growth factor; NGF: nerve growth factor; BDNF: brain-derived neurotrophic factor; PGE2: prostaglandin E2; TAC: total antioxidant capacity; 8-OHdG: 8-hydroxydeoxyguanosine.

## Data Availability

Data used in this study are available upon request from the corresponding author.

## Supplementary Materials

The following supporting information can be downloaded at: https://www.mdpi.com/2227-9059/11/3/673/s1, Table S1: The AUC of the COV of urinary biomarkers in discriminating different OAB subgroups and controls.
