# Supplementary material for: Discriminating Different Bladder and Bladder Outlet Dysfunctions by Urinary Biomarkers in Women with Frequency–Urgency Syndrome
Source: Biomedicines. 2023 Feb 23;11(3):673. doi: 10.3390/biomedicines11030673 (PMC10045187; doi:10.3390/biomedicines11030673)
Supplement: Supplementary file 1 [file biomedicines-11-00673-s001.zip › biomedicines-2202969-supplementary.pdf]

**Table S1.** The AUC of the COV of urinary biomarkers in discriminating different OAB subgroups and controls.

| HSB vs. Controls | AUC | COV | Sensitivity | Specificity | PPV | NPV |
|---|---|---|---|---|---|---|
| **IL-1β** | 0.584 | ≧ 0.440 | 85.2% | 48.5% | 57.5% | 80% |
| **IL-2** | 0.722 | ≦ 0.775 | 89.3% | 54.5% | 62.5% | 85.7% |
| **IL-6** | 0.564 | ≧ 0.385 | 100% | 18.2% | 50.9% | 100% |
| **IL-8** | 0.580 | ≧ 93.295 | 21.4% | 100% | 100% | 60% |
| **TNF-α** | 0.570 | ≧ 0.770 | 55.6% | 63.6% | 55.6% | 63.6% |
| **VEGF** | 0.684 | ≦ 3.410 | 46.4% | 97.1% | 92.9% | 68.8% |
| **NGF** | 0.659 | ≦ 0.245 | 65.5% | 61.8% | 59.4% | 67.7% |
| **BDNF** | 0.519 | ≦ 0.415 | 24.1% | 100% | 100% | 60% |
| **PGE2** | 0.581 | ≧ 180.605 | 48.3% | 75.8% | 63.6% | 62.5% |
| **8-isoprostane** | 0.612 | ≧ 22.610 | 41.4% | 81.8% | 66.7% | 61.4% |
| **TAC** | 0.776 | ≦ 318.66 | 58.6% | 91.2% | 85% | 72.1% |
| **8-OHdG** | 0.517 | ≦ 14.775 | 62.1% | 58.8% | 56.3% | 64.5% |
| **IDO + NDO + DV vs. HSB** | | | | | | |
| **IL-1β** | 0.693 | ≧ 0.610 | 66.4% | 81.5% | 93.8% | 36.7% |
| **IL-2** | 0.729 | ≦ 0.390 | 55.6% | 100.0% | 100.0% | 35.0% |
| **IL-6** | 0.510 | ≧ 1.650 | 32.5% | 85.7% | 90.2% | 23.8% |
| **IL-8** | 0.546 | ≧ 1.860 | 85.0% | 32.1% | 83.5% | 34.6% |
| **TNF-α** | 0.742 | ≧ 1.035 | 64.6% | 88.9% | 96.1% | 37.5% |
| **VEGF** | 0.547 | ≧ 3.235 | 73.7% | 46.4% | 84.8% | 30.2% |
| **NGF** | 0.598 | ≦ 0.115 | 28.4% | 100.0% | 100.0% | 25.9% |
| **BDNF** | 0.549 | ≦ 0.310 | 21.1% | 100.0% | 100.0% | 24.4% |
| **PGE2** | 0.557 | ≧ 133.01 | 72.8% | 44.8% | 83.8% | 29.5% |
| **8-isoprostane** | 0.545 | ≦ 10.19 | 37.9% | 79.3% | 88.0% | 24.2% |
| **TAC** | 0.681 | ≧ 316.32 | 75.9% | 58.6% | 87.6% | 38.6% |
| **8-OHdG** | 0.675 | ≧ 24.13 | 57.8% | 75.9% | 90.5% | 31.0% |
| **IDO + NDO vs. DV** | | | | | | |
| **IL-1β** | 0.710 | ≦ 0.695 | 69.6% | 75.0% | 81.4% | 61.1% |
| **IL-2** | 0.732 | ≧ 0.200 | 76.4% | 62.2% | 76.4% | 62.2% |
| **IL-6** | 0.707 | ≧ 0.885 | 66.2% | 69.8% | 78.3% | 55.6% |
| **IL-8** | 0.558 | ≧ 7.505 | 56.5% | 56.8% | 67.2% | 45.5% |
| **TNF-α** | 0.552 | ≧ 1.545 | 45.7% | 93.0% | 91.4% | 51.3% |
| **VEGF** | 0.742 | ≧ 9.080 | 61.4% | 88.6% | 89.6% | 59.1% |
| **NGF** | 0.619 | ≦ 0.125 | 45.8% | 100.0% | 100.0% | 53.0% |
| **BDNF** | 0.745 | ≦ 0.510 | 65.2% | 86.7% | 88.2% | 61.9% |
| **PGE2** | 0.682 | ≧ 185.24 | 67.1% | 68.2% | 77.0% | 56.6% |
| **8-isoprostane** | 0.771 | ≧ 17.745 | 61.1% | 84.1% | 86.3% | 56.9% |
| **TAC** | 0.541 | ≧ 1203.74 | 27.5% | 93.0% | 86.4% | 44.4% |
| **8-OHdG** | 0.578 | ≦ 23.59 | 49.3% | 71.1% | 72.9% | 47.1% |
| **IDO vs. NDO** | | | | | | |
| **IL-1β** | 0.758 | ≦ 0.510 | 58.1% | 89.5% | 81.8% | 72.3% |
| **IL-2** | 0.907 | ≧ 0.480 | 100.0% | 78.0% | 77.5% | 100% |
| **IL-6** | 0.544 | ≦ 1.600 | 74.2% | 50.0% | 53.5% | 71.4% |
| **IL-8** | 0.528 | ≧ 3.710 | 87.1% | 36.8% | 52.9% | 77.8% |
| **TNF-α** | 0.852 | ≦ 1.460 | 90.3% | 76.9% | 75.7% | 90.9% |
| **VEGF** | 0.682 | ≧ 7.850 | 90.3% | 56.4% | 62.2% | 88% |
| **NGF** | 0.894 | ≧ 0.130 | 100.0% | 80.5% | 79.5% | 100.0% |
| **BDNF** | 0.797 | ≧ 0.365 | 100.0% | 68.4% | 72.1% | 100.0% |

| | | | | | | |
|---|---|---|---|---|---|---|
| **PGE2** | 0.662 | ≦ 626.195 | 96.8% | 33.3% | 54% | 92.9% |
| **8-isoprostane** | 0.511 | ≧ 7.115 | 93.5% | 14.6% | 45.3% | 75.0% |
| **TAC** | 0.789 | ≧ 642.62 | 72.4% | 75.0% | 67.7% | 78.9% |
| **8-OHdG** | 0.523 | ≦ 38.155 | 77.4% | 35.0% | 48.0% | 66.7% |

Abbreviations: IDO: idiopathic detrusor overactivity; NDO: neurogenic detrusor overactivity; DV: dysfunctional voiding; HSB: hypersensitive bladder; IL: interleukin; TNF-$\alpha$: tumor necrosis factor-$\alpha$; VEGF: vascular endothelial growth factor; NGF: nerve growth factor; BDNF: brain-derived neurotrophic factor; PGE2: prostaglandin E2; TAC: total antioxidant capacity; 8-OHdG: 8-hydroxydeoxyguanosine; AUC: area under the curve; COV: cut-off value; PPV: positive predictive value; NPV: negative predictive value.
